# The Weight of Bariatric Surgery: Wernicke–Korsakoff Syndrome after Vertical Sleeve Gastrectomy—A Case Series

**DOI:** 10.3390/jpm14060638

**Published:** 2024-06-14

**Authors:** Melissa Gutiérrez-Rey, Lily Castellar-Visbal, Kaleb Acevedo-Vergara, José Vargas-Manotas, Diego Rivera-Porras, Gloria Londoño-Juliao, Brenda Castillo-Guerrero, María-Camila Perdomo-Jiménez, Valmore Bermúdez

**Affiliations:** 1 Universidad Simón Bolívar, Facultad de Ciencias de la Salud, Barranquilla 080001, Colombia; melissa.gutierrez1@unisimon.edu.co (M.G.-R.); lily.castellar@unisimon.edu.co (L.C.-V.); jose.vargas@unisimon.edu.co (J.V.-M.); gloria.londono@unisimon.edu.co (G.L.-J.); brenda.castillo@unisimon.edu.co (B.C.-G.); maria.perdomo@unisimon.edu.co (M.-C.P.-J.); 2 Clínica la Misericordia Internacional, Barranquilla 080001, Colombia; kalebacevedo@gmail.com; 3 Universidad Simón Bolívar, Facultad de Ciencias Jurídicas y Sociales, Centro de Investigación en Estudios Fronterizos, Cúcuta 540001, Colombia; diego.rivera@unisimon.edu.co

**Keywords:** anterograde amnesia, beriberi, cognitive disorder, neuropsychiatry, vertical sleeve gastrectomy, Wernicke–Korsakoff syndrome

## Abstract

In this case series, the simultaneous occurrence of Wernicke’s encephalopathy (WE) and dry beriberi was reported in three patients who underwent vertical sleeve gastrectomy (VSG) between May 2021 and May 2023. All patients were obese women who underwent vertical sleeve gastrectomy (VSG) without immediate postoperative complications, but two weeks later, hyperemesis and subsequent encephalopathy with ocular movement abnormalities and weakness were observed over the following thirty days. Patients were referred to neurology, where due to the high suspicion of WE, thiamine replacement therapy was initiated; meanwhile, diagnostic neuroimaging and blood tests were conducted. Neurological and psychiatric evaluations and neuroconduction studies were performed to assess the clinical evolution and present sequelae. One year after diagnosis, all patients exhibited affective and behavioral sequelae, anterograde memory impairment, and executive functioning deficits. Two patients met the criteria for Korsakoff syndrome. Additionally, peripheral nervous system sequelae were observed, with all patients presenting with sensorimotor polyneuropathy. In conclusion, Wernicke’s encephalopathy requires a high diagnostic suspicion for timely intervention and prevention of irreversible sequelae, which can be devastating. Therefore, raising awareness among medical professionals regarding the significance of this disease is essential.

## 1. Introduction

Micronutrient deficiencies (M.Ds.) have been extensively studied since the progressive discovery of vitamin structure and biological function in the early 20th century [1]. Once prevalent entities caused by food scarcity due to droughts, pandemics, and wars [2,3,4], avitaminosis became a major research focus [5], and through decades of research efforts, significant advancements had been made to develop strategies like food reinforcement [6].

It is important to highlight that M.D. is not only associated with inadequate dietary intake [7]. In developed countries, this landscape is commonly observed in alcoholism, chronic diseases, pregnant women [8,9], illicit substance users, patients on parenteral nutrition, chronic diuretic therapy, and in post-bariatric surgery (B.S.) patients [10,11].

B.S. significantly reduces the body’s ability to absorb nutrients, even with a healthy diet. This effect is due, at least in part, to a reduction in the stomach volume, since both laparoscopic sleeve gastrectomy (LVSG) and Roux-en-Y gastric bypass (RYGB) involve a significant reduction in stomach size: Firstly, in LVSG, a large portion of the stomach is resected, while in RYGB, a small gastric pouch is created, limiting food intake and restricting the gastric digestion phase. Secondly, RYGB specifically bypasses (rerouted) food from the stomach pouch directly to the distal jejunum, bypassing the duodenum and proximal jejunum, which are crucial for nutrient uptake. Consequently, these mechanisms can lead to deficiencies in vitamins B1, B12, C, D, folate, A, K, and minerals like iron, selenium, zinc, and copper essential vitamins and minerals, highlighting the importance of close monitoring and tailored supplementation regimens, starting when a blended food stage begins. Regrettably, over-the-counter supplements may not provide enough vitamin B12, thiamine, iron, or fat-soluble vitamins, so higher doses may be needed. For this reason, it is not surprising that post-B.S. patients manifest neurological impairments like peripheral neuropathy, Wernicke encephalopathy (WE), and Korsakoff psychosis [12,13].

In this context, thiamine deficiency (T.D.) has been addressed in patients who have undergone B.S. in several studies, reporting a 10.2–12.3% prevalence after RYGB [9,12] and 5–25% prevalence for LVSG [14], a very different panorama when compared to the 1.5% T.D. prevalence observed in the general population [15]. Moreover, a recent meta-analysis by Karimi et al. found a pooled T.D. prevalence of 7% (95% CI: 4–12%) at baseline, but T.D. prevalence at three, six, and twelve months after surgery was 19%, 9%, and 6%, respectively [16]. It must be addressed that only 3 of 41 studies in this meta-analysis were carried out in developing countries; therefore, these results cannot be extrapolated to middle- and low-income countries where T.D. prevalence is expected to be higher [16].

Therefore, strict adherence to pre- and postoperative management guidelines, coupled with rigorous long-term follow-up, is paramount in preventing B.S. complications [17,18,19]. Unfortunately, there is a paucity of real-world data studies assessing the consultations number and the quality of care offered by healthcare providers, as well as nutritional follow-up and adherence to multivitamin therapy monitoring. This presents a significant obstacle to analyzing patient progress and, consequently, capturing relevant clinical data in post-bariatric patients [20,21]. This landscape becomes even more intricate in the absence of centralized national records and limited access to comprehensive medical records encompassing long-term patient outcomes.

This paper aims to describe a case series of three patients who, following LSVG, developed a spectrum of thiamine deficiency manifestations. These manifestations include dry beriberi, Wernicke’s encephalopathy (WE), and Korsakoff’s psychosis. In addition, the evolution of neurological, cognitive, and psychiatric symptoms in these patients is detailed. The relatively frequent occurrence of ET after BD is emphasized, highlighting the importance of early diagnosis and prompt treatment of this condition. Finally, it is worth mentioning that approximately 1884 bariatric surgeries are performed in our center per year, which underlines the relevance of this study in the current context.

## 2. Case 1

A 30-year-old woman with a morbid obesity diagnosis (BMI: 43 Kg/m^2^) (Table 1), polycystic ovary syndrome, and amenorrhea underwent LVSG on 17 August 2021 after failing lifestyle interventions that included exercise, a low-calorie diet, and follow-up by a nutrition. Two weeks after surgery, during her transition from a liquid to a solid diet, recurrent vomiting and abdominal pain led to emergency room visits on two consecutive days. She was treated with intravenous fluids and antiemetics (metoclopramide). During these visits, no neurological deficits were observed. On 3 September 2021, she transitioned from vomiting on each meal to vomiting around once a day, with vomiting exacerbations and oral intolerance that led her to another two emergency room visits. On 22 September 2021, during the last emergency admission, the patient underwent an upper gastrointestinal endoscopy that reported grade II gastroesophageal reflux. From October, the vomiting persisted once daily. By November 19th, she developed progressive myalgia, weakness, and paresthesia in her extremities associated with progressive gait limitation, leading her to the emergency room on 1 December 2021, being evaluated by a gastroenterologist, who ordered 1 mg of intramuscular cyanocobalamin. Due to her torpid clinical evolution, the patient was hospitalized and requested a neurology consultation.

**Figure 1:**
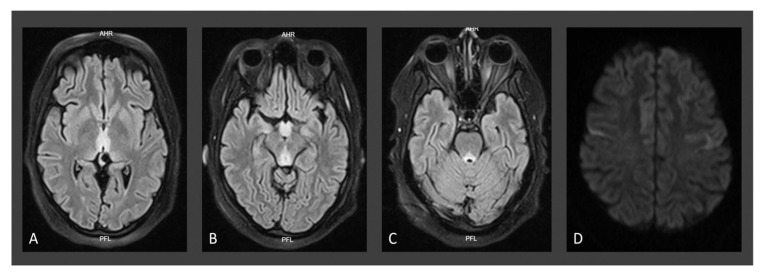
MRI findings in case 1: axial T2-weighted FLAIR image showing symmetrical hyperintensity in thalamus adjacent to the third ventricle (**A**); periaqueductal mesencephalic region, mesencephalic tectum, mammillary bodies (**B**) and dorsal region of the pons (**C**) associated with diffusion sequence restriction in these areas (not shown) and in frontoparietal cortex (**D**).

Initial neurological evaluation revealed somnolence, and despite being easily alerted, she was not able to speak or obey orders. Vertical gaze palsy, multidirectional nystagmus, allodynia, weakness (muscle strength 3/5 in the upper limbs and 2/5 in the lower limbs), and absent reflexes were evidenced (Table 2). Given the clinical picture that was highly suggestive of WE, free plasma thiamine and erythrocyte transketolase activity were requested, and intravenous thiamine and oral folic acid were administered to ensure prompt treatment (Table 3). Laboratory tests showed moderate normocytic normochromic anemia (hemoglobin 9.7 g/dL, N.R.: 11.2–15.7), decreased folate levels (3.2 ng/mL, N.R.: 4.6–34.9 ng/mL), and elevated cyanocobalamin (2000 pg/mL, N.R.: 174–878 pg/mL), explained by recent administration of this vitamin. Regrettably, thiamin tests could not be performed due to health insurance company problems. Simple and contrast-enhanced brain MRI showed typical WE findings (12 April 2021) (Figure 1).

Twelve hours after starting treatment, the patient presented with marked improvement in consciousness, but bradypsychia and bradylalia were still present, enabling the opportunity for dialog with the patient, noting anterograde amnesia, as she could not remember recent events.

On the day of discharge (12 June 2021, 15 days after starting treatment), there was no improvement in bradypsychia, multidirectional nystagmus, weakness in all four extremities, or areflexia. The patient also began to present feelings of sadness, which became more pronounced over time. Extremity electromyography and nerve conduction studies showed findings compatible with sensory and motor polyneuropathy with myelinic and axonal involvement of all extremities, thus confirming a dry beriberi diagnosis (Table 3).

Six months later, the patient could take her first assisted steps, and two years later, she exhibited full strength recovery in her arms and persistent muscle weakness in both legs; therefore, she is currently using a walker to assist with ambulation. Along with this, the nystagmus is still ongoing (Table 2). During the neuropsychological examination, she reported sadness, anhedonia, irritability, worthlessness and hopelessness, passive death ideation, and affective sphere loss and functionality, thus configuring a major depressive disorder without psychotic symptoms, and the functional evaluation revealed mild cognitive impairment in both working memory and cognitive flexibility. She is currently on aline (100 mg/day) and intensive cognitive behavioral therapy (CBT).

## 3. Case 2

A 22-year-old woman with morbid obesity (BMI: 43.3 Kg/m^2^) (Table 1) underwent VSG on 16 July 2022 after failing to achieve weight loss goals in an obesity management program. Before surgery, the patient actively participated in their healthcare provider’s obesity management program, which included psychological assessment, gym attendance, and nutritional counseling, with no further nutritional laboratory assessment or vitamin supplementation.

The immediate postoperative period was uneventful, and in the first 15 days following surgery, she had no issues with liquid diet tolerance. The patient additionally reported that she did not receive nutritional follow-up or post-surgical multivitamin therapy following the surgery. This information was corroborated by her family members. Three weeks later, at the end of the liquid–solid diet transition, she experienced anorexia, vomiting, and oral route intolerance. She consulted the emergency department six times between August 6th and September 8th, receiving intravenous hydration and antiemetic therapy (metoclopramide and ondansetron) without improvement. On 9 September 2022, she developed lower-limb weakness and cramps, and four days later, she looked for medical attention from a gastroenterologist. He observed anxiety and emotional lability and referred her to outpatient psychiatry and neurology consultation. It is important to note that psychological evaluation before surgery was normal. However, on 17 September, a psychiatric evaluation revealed anxiety, irritability, and sleep-onset insomnia, for which pharmacological management with fluoxetine (20 mg qd) and zopiclone (7.5 mg qd) was initiated. It is important to note that on the same day during the neurology consultation, no clear diagnosis was made requesting serum thiamine concentrations, cyanocobalamin, and peripheral neural conduction studies.

One day later, the conditions worsened, adding dyspnea and chest pain. The patient decided to consult the emergency department again. On admission, the patient’s vital signs were stable. She was initially evaluated by internal medicine, who, due to suspicion of vitamin deficiencies, began treatment with cyanocobalamin (1000 mcg I.M. daily for seven days), and a neurology assessment was requested. During the same day, a comprehensive neurological examination was made, revealing somnolence, bradypsychia, anterograde amnesia, vertical nystagmus, severe hearing loss, vertigo, and both lower (1/5) and upper (4+/5) extremity weakness, alongside with upper extremity hyporeflexia and lower extremity areflexia with tenderness on palpation (Table 2).

**Figure 2:**
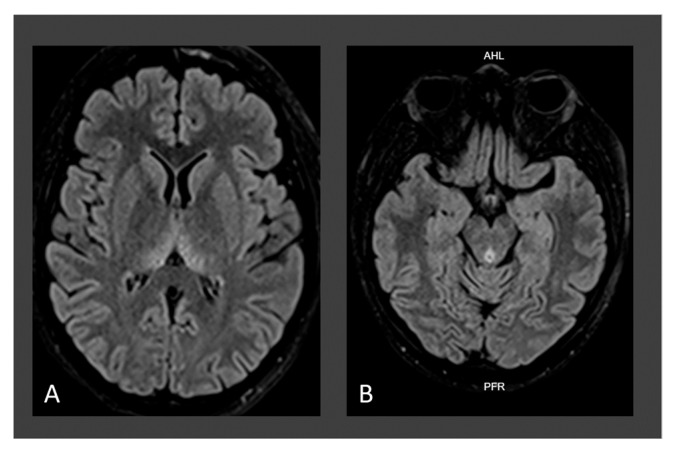
Brain MRI findings of case 2: axial FLAIR T2-weighted image showing symmetrical hyperintensity in thalamus adjacent to the third ventricle (**A**) and periaqueductal mesencephalic region (**B**) associated with diffusion sequence restriction in these areas (not shown).

Given both Wernicke encephalopathy and dry beriberi suspicion, baseline thiamine levels and erythrocyte transketolase were requested, but they were not processed due to administrative problems with the health insurance company. Folate levels were decreased (3.3 ng/mL, N.R.: 4.6–34.9 ng/mL), and cyanocobalamin was elevated due to recent administration. Simple and contrast-enhanced brain MRI (23 September 2022) showed changes consistent with Wernicke’s encephalopathy (Figure 2). Intravenous thiamine treatment was initiated (Table 3). On the fourth day of treatment, the patient’s bradypsychia improved; however, anterograde amnesia, vertical nystagmus, hearing loss, and muscle weakness persisted until discharge.

Seven months later, her strength improved substantially. However, she required a walker aid due to vertigo, persistent left tinnitus, and burning pain in her lower limbs, which was managed with pregabalin. Nerve conduction studies showed severe lower-limb sensorimotor polyneuropathy with myelinic and axonal involvement. Tonal audiometry revealed mild bilateral mixed hearing loss. After one year, there was an improvement in bradypsychia, but the anterograde amnesia persisted. It is important to note that learning was difficult from lecture material, evidenced by low performance in short-term visuospatial memory tasks and significant weaknesses in the short-term verbal memory factor. Additionally, cognitive flexibility decline and inhibition control factors were observed in the Trail Making Test Part B and Stroop test tasks (Table 4), translating into metacognitive and behavioral dysexecutive syndrome. In addition to the findings mentioned above, emotional lability, anxiety, sleep problems, and intrusive thoughts interrupted her daily activities; therefore, the pharmacological treatment was modified to eszopiclone, with an increase in pregabalin dose.

## 4. Case 3

A 28-year-old woman with morbid obesity (BMI: 34.9 Kg/m^2^) (Table 1) who was unable to achieve weight control goals underwent a VSG on 15 July 2022, without immediate complications. It is important to highlight that the patient and her family members both reported that she did not undergo laboratory nutritional assessment or receive multivitamin therapy before surgery. Following surgery, she received weekly nutritional follow-up for three weeks. Still, after the change of diet consistency from liquid to bland foods (approximately 17 days post-surgery), she experienced repetitive emetic episodes, leading to two instances of at-home treatment with intravenous fluids and antiacids (omeprazole, 40 mg BID) with no improvement given the persistence of the daily emetic episodes. The patient received both a daily oral multivitamin tablet and a 900 mcg biotin tablet during the first postoperative month. However, she only tolerated this treatment for 30 days due to daily emetic episodes (at a frequency of 1–3 episodes per day).

**Figure 3:**
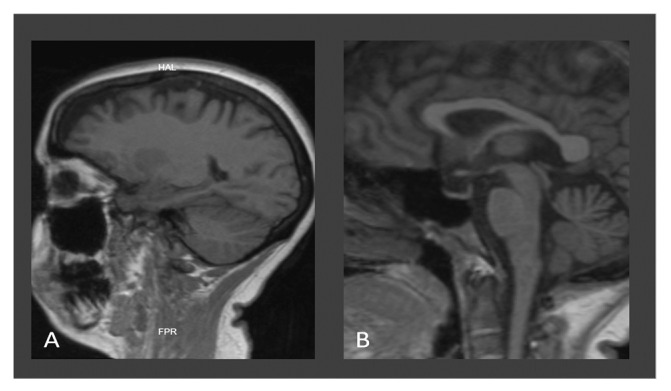
Brain MRI findings of case 3: Sagittal T1-weighted image showing frontal (**A**) and mammillary body (**B**) atrophy.

On 3 September 2022, her emetic episodes continued but were once a day, but not every day; she developed progressive gait difficulty, tinnitus, and amnesia regarding recent events, prompting a visit to the emergency department five days later to La Merced Clinic. On admission, the patient displayed stable vital signs, drowsiness, anterograde amnesia, nystagmus, right-sided tinnitus, and hyperacusis. Neurological examination revealed weakness in all extremities, areflexia, and decreased sensitivity to vibration and fine touch of the lower limbs (Table 2). Laboratory results indicated hypokalemia and deficiencies in folic acid and cyanocobalamin, prompting replacement therapy, to which she responded with strength improvement in the upper extremities throughout the days. A simple cerebral MRI revealed typical findings of Wernicke’s encephalopathy (Figure 3), leading to intravenous thiamine treatment on 21 September 2022, and the day after, she was referred to our institution for treatment continuation (Table 3).

On the day of admission, the patient had improved strength in the upper (5/5) and lower extremities (2/5), with normal reflexes in the upper extremities and areflexia in the lower extremities. Still, anterograde amnesia, nystagmus, tinnitus, and hyperacusis persisted. The potassium, cyanocobalamin, and folic acid levels were back to normal. As the treatment continued, the blood thiamine concentration returned to normal after several days, but unfortunately, no serum thiamine concentration test was requested before initiating therapy. Upon discharge, she completed four days of intravenous thiamine replacement therapy and was no longer drowsy, and her tinnitus slightly improved. She continues oral thiamine supplementation to this day.

One year post-diagnosis, neurological examination demonstrated significant improvement in strength and sensory deficits, with persistent nystagmus (Table 2). Nerve conduction studies revealed severe sensorimotor polyneuropathy with myelinic and axonal involvement of the lower limbs. The neuropsychological evaluation indicated cognitive impairment in complex attention factors, cognitive information processing speed, and short-term verbal and visuospatial memory with anterograde amnesia. Additionally, there were executive function disturbances, working memory factors, and cognitive flexibility alterations (Table 4), indicative of metacognitive and behavioral dysexecutive syndromes. A comprehensive psychiatric assessment revealed a personality disorder with maladaptive behavioral patterns and inhibition control issues, which she previously had, as well as metacognitive component changes, irritability, and indifference, which made her reluctant towards therapy and also worsened her interpersonal relationships.

## 5. Discussion

Obesity is a global public health issue [22]. In Colombia, 56% of the population is overweight and 19% is obese, affecting approximately 10 million people [23], with a significantly higher prevalence in lower socioeconomic strata [24]. Given the strong association of obesity with cardiovascular risk [25], various strategies have been developed to address this increasingly prevalent condition. These strategies include lifestyle changes, psychotherapy, pharmacological interventions, and bariatric surgery.

Substantial clinical evidence supports bariatric surgery (B.S.) because, in contrast to nonsurgical interventions [26], B.S. yields superior outcomes in weight loss [27], quality of life [28], blood pressure control [29], acute cardiovascular event prevention [30,31], better plasma glucose control in patients with type 2 diabetes [32], and even diabetes reversion [33], both in short- and long-term follow-up [34,35]. Moreover, a recent meta-analysis of 20 studies found a statistically significant improvement in immediate verbal memory and long-term memory in patients treated with RYGB, as opposed to those undergoing sleeve gastrectomy, which was not associated with significant changes in cognitive function [36].

These outcomes have triggered a significant shift in B.S., transitioning from a last-resort option to being acknowledged as the most effective approach in managing both morbid obesity and grade II obesity with associated comorbidities [37]. Thus, in the past four decades, there has been a remarkable 30-fold surge in bariatric surgery procedures worldwide [38]. In this regard, Colombia has not been immune to the rapid growth in bariatric procedure trends, and its healthcare system has adapted to offer this therapeutic alternative within the Mandatory Health Plan, making it accessible to individuals with limited financial resources.

In this regard, the non-reversibility of these surgical techniques provides lifelong benefits but entails continuous medical follow-up because, despite meticulous surgical techniques, complications can arise due to shifts in nutrient absorption and signaling pathway alteration, resulting in nutritional deficiencies. Multiple factors contribute to the heightened risk of micronutrient deficiencies after bariatric surgery. RY-GB and VSG involve the removal of a portion of the stomach, with RY-GB additionally bypassing the duodenum and proximal jejunum [39]. Since the stomach and small bowel are integral to micronutrient absorption, these procedures can impact absorption efficiency [40]. Overall, bariatric procedures reduce oral intake and dietary micronutrient consumption [41], increasing the risk of vitamin deficiencies, especially when proper supplementation is not ensured [42].

As a result, some clinical practice guidelines advocate for regular monitoring of micronutrients and lifelong vitamin and mineral supplementation in post-bariatric follow-up, but regrettably, such recommendations are based on limited data [43]. Additionally, routine micronutrient monitoring and supplementation costs may hinder widespread clinical implementation. Consequently, there is a pressing need for a more comprehensive understanding of micronutrient deficiency prevalence and postoperative micronutrient supplementation patterns [44]. In this regard, thiamine deficiency is a concerning issue for post-bariatric patients because of its frequency (18% to 49% in certain studies) and the burden of its neurological sequelae [45,46].

Thiamine’s importance in higher organisms is due to the extensive enzyme array in which its coenzyme, thiamine pyrophosphate, is found. Over 20 enzymes (from prokaryotes and eukaryotes) dependent on thiamine pyrophosphate are recognized. Noteworthy examples include glyoxylate carboligase [47], transketolase [48], phosphoenolpyruvate decarboxylase [49], 2-hydroxyacyl-CoA lyase [50], pyruvate dehydrogenase complex [45], 2-hydroxyphytanoyl-CoA lyase [51], branched-chain amino acid enzyme [52], phosphoketolase [53], benzoyl formate decarboxylase, acetohydroxyacid synthase [54], pyruvate decarboxylase [55], 2-oxoglutarate dehydrogenase complex [56], sulfopyruvate decarboxylase [57], pyruvate ferredoxin oxidoreductase [58], and phenylpyruvate decarboxylase [59].

Archaea, bacteria, algae, plants, protozoans, and some fungi have metabolic pathways for de novo thiamine synthesis [60]. This molecule is an exogenous substance for all vertebrates and must be incorporated through food. One hypothesis to explain the inability of animals to synthesize thiamine posits that the ubiquity of this vitamin leads to a lack of its production [61]. Another hypothesis is that thiamine synthesis increases energy costs and metabolic needs because it requires significant energy and resources, which can be metabolically expensive for animals. By relying on external sources of thiamine, animals can conserve energy and resources, which can be utilized for other metabolic processes [62]. Thiamine phosphate esters derived from dietary sources undergo hydrolysis, resulting in the formation of thiamine or ThMP by intestinal alkaline phosphatase catalysis [63]. Subsequent transportation across the brush border membrane occurs predominantly in the upper jejunum by active and passive mechanisms [64]. Active absorption occurs when thiamine intraluminal concentrations are low by electroneutral antiport thiamine transporters-1 and 2 (hTHTR-1, hTHTR-2); an outwardly directed proton gradient activates this high-affinity transport mechanism (km < 1 mM) [65].

Conversely, passive transport seems to proceed at high intraluminal thiamine concentrations. Once portal circulation is reached, free thiamine undergoes active translocation to the liver, erythrocytes, and white blood cells, harboring approximately 90% of the total bloodstream thiamine [66]. Nevertheless, free-circulating thiamine undergoes excretion by the kidneys, with a rate loss strongly associated with renal clearance; thus, diuretic therapy may contribute to thiamine deficiency in cardiovascular patients [67].

In mammals, thiamine pyrophosphate is an enzymatic cofactor involved in three crucial metabolic processes [68]. At first, this molecule plays a pivotal role in the catalytic function of numerous enzymes in the bioenergetics pathways [69] and amino acid [52] and carbohydrate metabolism, including pentose synthesis, crucial for both nucleotide synthesis and NADPH + H^+^ production for antioxidative defense [47,70]. Secondly, thiamine-phosphorylated derivatives act as allosteric regulators [71], as well as being involved in nervous transmission and environmental stimulus reception signaling [72]. Finally, the compelling findings from numerous studies strongly suggest that thiamine, its phosphorylated derivatives, and thiamine-dependent enzymes play a crucial role in environmental factor responses to oxidative stress and pathogens [73,74].

Thiamine has a relatively short half-life, ranging from 1 to 12 h; thus, this nutrient’s storage in the body is limited to 1 to 3 weeks. Therefore, regular thiamine consumption is essential to ensure adequate intracellular levels [75]. Thiamine deficiency in humans leads to disruptions in many key processes, including glucose metabolism and both pyruvate and alpha-ketoglutarate oxidative decarboxylation in the Krebs cycle, resulting in reduced ATP synthesis, lactic acidosis, and mitochondrial dysfunction [76]. Insufficient DNA synthesis can also be observed because of low transketolase activity and ribose-5-phosphate synthesis, resulting from decreased pentose phosphate pathway activity. Consequently, neurotransmitter synthesis from glucose-derived amino acids is disrupted [66]. Functional impairment in P.D. and α-KGDH increases lactate and pyruvate within a week, causing functional disruptions in nerve cells due to decreased ion pump activity and secondary edema. After 7–10 days, deficiency in erythrocyte transketolase and increased nitric oxide due to endothelial dysfunction resulted in low intracellular glutamate levels, elevated extracellular glutamate levels, loss of osmotic gradients, and an increase in free radical production and proinflammatory cytokine release, leading to cytotoxic and vasogenic edema. Finally, after 14 days of thiamine deficiency, there is DNA fragmentation, neuronal necrosis, and irreversible brain injury [77,78].

This phenomenon underscores the importance of timely treatment initiation to prevent permanent neurological sequelae [37]. In this regard, T.D. usually occurs in three scenarios: by increased requirements (e.g., malignancy, sepsis), increased losses (e.g., hemodialysis, diuretic therapy, hyperemesis), or reduced absorption/insufficient apporting (e.g., alcoholism, bariatric surgery, malnutrition) [79,80]. In this context, we describe three bariatric surgery patients and T.D. presenting with beriberi, WS, and K.S.

Regrettably, the initial T.D. symptoms are nonspecific and can easily be attributed to various pathological processes. Relentless or unusual fatigue and mood changes with a tendency towards hyperirritability and mood lability are common [81]. A sense of mental confusion and subtle decreases in memory, appetite loss, sleep disturbances, gastrointestinal (G.I.) discomfort, and dysmotility are frequently reported. As deficiency progresses, food intolerance and vomiting may develop. Both experimental studies [81] and clinical cases [82] suggest that gastrointestinal discomfort and dysmotility may be the earliest thiamine deficiency symptoms, and they are more prevalent than currently accepted. In our clinical cases, all three patients had gastrointestinal symptoms. Case 1’s initial symptoms were abdominal pain associated with hyperemesis, which led her to recurrent emergency visits, and cases 2 and 3 had not only hyperemesis but also an associated lack of appetite before the instauration of neurological symptoms.

All patients included in this case series were young women with morbid obesity without an alcoholism history. Following VSG, all patients developed hyperemesis, along with a clinical picture of lower motor neuron injury characteristic of dry beriberi polyneuropathy. A length-dependent sensorimotor alteration with abolished reflexes and predominantly positive sensory symptoms was observed (Table 1). Furthermore, all patients exhibited encephalopathy, but only one experienced ophthalmoplegia, and none showed ataxia, indicating that the classic triad of Wernicke’s disease was not detected. However, it is essential to note that, upon admission, all patients had generalized weakness and drowsiness, complicating the comprehensive assessment of ataxia [83,84] (see Table 2). These facts led to a presumption of thiamine deficiency diagnosis. In this regard, some studies point out the wide clinical presentation of T.D., ranging from the classic triad of encephalopathy, ophthalmoplegia, and ataxia observed in only 16–33% of cases [37] to vestibular nucleus deficits and posterior hypothalamus involvement leading to hypotension, tachycardia, syncope, and respiratory distress [85].

Dry beriberi and WE diagnoses are primarily clinical. From a theoretical point of view, prompt risk factor identification must guide a proper diagnosis; however, in the absence of traditional risk factors such as malnutrition, alcoholism, or severe gastrointestinal discomfort or illness, these symptoms’ similarity to those of other conditions complicates an early clinical diagnosis [83,86]. Consequently, conventional descriptions of T.D. primarily focus on the late manifestations observed in wet beriberi, dry beriberi, and Wernicke’s and Korsakoff’s encephalopathies, categorizing symptoms based on specific organ involvement. For example, high-output heart failure and edema are associated with wet beriberi; peripheral neuropathies, muscle pain, and weakness are associated with dry beriberi; and the classic neurological triad of mental confusion, ocular abnormalities, and ataxia are associated with WE [86]. In this context, a study based on 131 cases of fatal WE revealed a concerning finding, as 80% of the cases were not diagnosed during the patient’s lifetime (likely because only 16% presented with the classic triad), 44% had one or two of the three symptoms, and 19% had only one [87]. A valuable tool for WE diagnosis is Caine’s criteria (Table 5), proposed by Caine et al. in 1997, with a sensitivity of 85% and a specificity of 100% in the alcoholic population. Because of the strong overlap, these criteria can be used for Korsakoff syndrome screening [88,89,90]. In our cases, the patients had two of the three required symptoms, which added to the history of malnutrition; they completed Caine’s criteria for WE.

Post-surgical management of these patients revealed deviations from the traditional protocol. First, there was irregular follow-up and a lack of preventive measures to avoid thiamine deficiency development, as none of the patients took thiamine-containing supplements in the first month, and postoperative gastroenterology follow-up appointments were scheduled for one month after surgery.

Additionally, despite seeking emergency medical care due to hyperemesis and neurological symptoms, acute T.D. was not suspected in the context of recent bariatric surgery history, and only cyanocobalamin supplements, intravenous fluids, and antiemetics were administered to manage the vomiting episodes. In this context, hyperemesis could be a risk factor for developing this disease or one of the first signs of clinical thiamine deficiency. However, considering the rapid development of symptoms, it is mandatory to consider other hypothetic precipitating factors that could have occurred individually or collectively: (1) patients may have had a pre-existing thiamine deficiency; (2) the postoperative diet may not have contained sufficient thiamine; (3) patients did not take oral supplements containing thiamine; and (4) food volumes were small and poorly designed in their composition, potentially causing a significant deficiency of this vitamin. In this regard, it has been reported that pre-existing micronutrient deficiencies are not uncommon in bariatric surgery candidates [91,92], and although the most frequent micronutrient deficiencies documented are vitamins B12 and D, folate, and iron, thiamine deficiency should not be underestimated, especially in the context of challenges in its determination.

To date, pre-existing deficiencies have been attributed to poor dietary quality, with low dietary micronutrient sources relative to caloric intake [92]. As thiamin preoperative screening does not yet form part of some bariatric surgical management guidelines [72], undetected and uncorrected deficiencies at baseline may exacerbate postoperative deficiency. Also, overseen T.D. commonly occurs in obese patients, as reported by Flancbaum et al., who found that T.D. occurrence in patients undergoing bariatric surgery rounds is between 15 and 29%, but not only that, they found that the prevalence was higher in African Americans (31%) and Hispanic people (47.2%) in comparison to Caucasian people (6.8%) [93].

Due to the lack of availability of highly sensitive tests for the quantification of thiamine or its metabolites or to measure the activity of erythrocyte transketolase [94], the diagnosis was made on a clinical basis and with the assistance of neuroimaging, particularly brain MRI, the preferred examination technique in these cases. All of our patients presented with neuroimaging findings that allowed for diagnostic confirmation [37]. In this regard, classical imaging changes are hyperintensity in the thalamus, third ventricle wall, periaqueductal grey matter, tectal plate, hypothalamus, and mammillary bodies, and with lower frequency in the cerebral cortex, fornix, corpus callosum splenium, caudate nuclei, red nuclei, cranial nerve nuclei, cerebellum, vermis, and dentate nucleus. In the chronic phase of the disease, enlargement of the third ventricle and atrophy of mammillary bodies are evident [10]. All patients presented typical findings on neuroimaging (Figure 1, Figure 2 and Figure 3), and in the case of patient 3, a follow-up MRI was performed one year later, revealing mamillary and frontal cortex atrophy, which is a chronically reported finding in the WE literature (Figure 3) [95].

Korsakoff syndrome (K.S.) is an irreversible stage of Wernicke’s disease, and its diagnosis follows the DSM-5 criteria for a major amnestic neurocognitive disorder with confabulation. It is characterized by anterograde amnesia and, to a lesser extent, retrograde amnesia for declarative knowledge. In addition, it is associated with executive deficits and anosognosia. Peripheral neuropathy is often present [96], but there are instances in which anterograde amnesia is the only element in K.S. Despite some isolated case reports, there is still no solid evidence for any specific treatment for cognitive impairment in K.S. patients [80,97]. However, it is worth noting that the absence of K.S. does not imply the absence of anterograde amnesia. Histopathological studies at the New South Wales Tissue Resource Center described a subgroup of patients with residual WE syndrome. This group was characterized by irreversible damage to various structures (mammillary bodies, thalamus, and other diencephalic structures) and residual cognitive dysfunction (especially executive and visuoconstructive dysfunction), without anterior thalamic nucleus damage, without severe memory dysfunction, and thus without K.S. [89]. In our cases, cases 2 and 3 had Korsakoff syndrome, and case 1 had residual cognitive syndrome with working memory sequelae without fulfilling the criteria for established Korsakoff syndrome (Table 4). In all three cases, peripheral neuropathy was confirmed by nerve conduction studies (Table 3).

There is no consensus on the optimal thiamine regimen in these cases; however, high doses of thiamine are recommended, generally 500 mg administered intravenously three times a day for a minimum of three days, followed by indefinite oral thiamine administration [98]. In our case series, all the patients showed rapid and marked improvement after thiamine administration. The first symptoms to improve were encephalopathy and ophthalmoplegia, in contrast to nystagmus, lower-limb muscle weakness, and occasional memory deficits that persisted over time (Table 2). The literature indicates that ocular abnormalities are the first to resolve after treatment initiation, including ophthalmoplegia, ptosis, hemorrhages, papilledema, anisocoria, and miosis, except for nystagmus, which is the most common ocular abnormality and may take months to resolve. In our study, all patients had persistent nystagmus a year after the diagnosis [80].

Despite receiving treatment with high doses of thiamine and subsequent supplementation throughout the year, it is crucial to highlight that our patients exhibited cognitive and psychiatric sequelae. Neuropsychological assessment revealed significant impairments in executive functioning in all three cases, in addition to the expected deficits in verbal short-term semantic memory and visuospatial short-term memory factors (Table 4). Executive functions are crucial skills that regulate and direct human behavior and cognition; thus, deficiencies in these skills lead to executive dysfunction syndromes that affect an individual’s ability to regulate behavior, emotions, and decision-making. The three cases showed suboptimal performance in tests associated with executive functioning, cognitive flexibility, working memory, and inhibition, such as the Trail Making Test Part B and Stroop test. In this regard, all three patients exhibited different psychiatric manifestations. Case 1 presented with a severe depressive disorder without psychotic symptoms; case 2 showed an organic anxiety disorder; and case 3 displayed an underlying previously undiagnosed personality disorder that evolved into increased maladaptive behavioral patterns, impulsivity, and metacognitive component impairments, consistent with the observed cerebral atrophy in the frontal lobe. These psychiatric sequelae adversely affect functionality, quality of life, and ability to maintain interpersonal relationships.

Considering the sequelae presented by patients suffering from the different manifestations of TD, which generate physical, cognitive, and psychiatric sequelae, it is critical to improve the preventive strategy for the different micronutrient deficiencies that can occur in patients undergoing BS.

Preoperative identification and correction of micronutrient deficiencies is a practice that should be considered in all patients before bariatric surgery, as some studies have suggested a relatively high prevalence of micronutrient deficits, as previously discussed. Thus, supplementation before surgery is faster and more effective than its management during the postoperative period [99]. However, clinical guidelines focus on micronutrients whose concentrations typically decrease during a long period of malabsorption or malnutrition (iron, calcium, vitamins B12, A, E, D, and K). However, unlike other micronutrients, they do not consider thiamine hypovitaminosis, which occurs early postoperatively and develops within days [100]. Currently, the clinical practice guidelines for the perioperative nutrition, metabolic, and nonsurgical support of patients undergoing BS in the USA state that the preventive measures for thiamine hypovitaminosis and micronutrient deficiencies should include preoperative screening for diabetes, dyslipidemia, kidney function, and nutritional deficiencies. Therefore, a blood count, ferritin, folate, vitamin B12, 25-hydroxyvitamin D, calcium, and parathyroid hormone measurements, liver function tests, and urea, electrolyte, fasting glucose, HbA1c, and lipid profiles should be requested [101]. Most centers recommend a preoperative low-calorie, low-carbohydrate diet to reduce the size of the liver to reduce surgical risk of liver laceration [102]. The European Association of Endoscopic Surgery (EASES) guidelines on bariatric surgery also support this practice [100].

In Colombia, the national guidelines that determine good practice regarding bariatric surgery perioperative assessment are the ACOCIB guidelines (Asociación Colombiana de Obesidad y Cirugía Bariátrica), last updated in 2018. In these guidelines, there is no standardized indication for measuring micronutrient levels before BS; after the surgery, only iron, ferritin, cyanocobalamin, calcium, and vitamin D measurements are indicated, and the rest of the micronutrients are tested according to clinical manifestations of deficiency [103]. In our three cases, both patients and relatives confirmed that there was no pre-surgical testing for micronutrient deficiencies.

During the early postoperative period, a liquid or very soft diet usually starts for the first few days after surgery to minimize regurgitation and vomiting. The consistency of the food is then gradually increased over the first few postoperative weeks. Before discharge, patients and their families should receive specific counseling from an experienced bariatric dietitian on this post-surgical meal progression and should also include clear instructions on warning signs and the potential risks of untreated hyperemesis [104]. After the dietary transition from liquids to solids, patients should be regularly evaluated by an experienced dietitian to prevent nutritional deficiencies and reduce the risk of weight regain [100,105]. In our three cases, only case 3 was followed closely by a nutrition within the first three weeks after the procedure, but in week 3, when she had nearly one week of hyperemesis, preventive micronutrient deficiency measures were not secured.

According to the 2017 European guidelines and the most recent American consensus, systematic supplementation with multivitamin complexes is recommended, considering the high incidence of hypovitaminosis, even years after surgery, and multivitamin supplements appear to reduce it. Among the micronutrients listed by the American Society for Metabolic and Bariatric Surgery (ASMBS), it is recommended to supplement at least 1.2 mg/day, a dose found in many of the current multivitamins [106]. It is important to note that patients who experience nausea, vomiting, or loss of appetite may require thiamine administration intramuscularly or intravenously. Additionally, it is essential to avoid administering dextrose-containing solutions without thiamine, as this can rapidly deplete thiamine stores [107]. In our cases, there was no micronutrient formulation at the hospital discharge, and regarding oral intolerance, in the ACOCIB guidelines, there is an indication of intravenous micronutrient supplementation in case of severe deficiency [103] but there is no mention of intravenous micronutrient supplementation in case of oral intolerance, as is mentioned in the British Obesity and Metabolic Surgery Society Guidelines [103,107].

Regarding the quality assessment of periprocedural measures, there is scarce information regarding the compliance of perioperative guidelines in Bariatric Surgery centers. Haythem Najah et al. conducted a study to evaluate the compliance of the guidelines regarding bariatric surgery in France, which are the French National Health Care Authority guidelines (Haute Autorité de Santé). The guideline compliance of 11,824 patients nationwide was analyzed after collecting data from the national database on patient characteristics, perioperative assessment, hospitalization, and postoperative follow-up from patients that had bariatric surgery in January 2014. It was found that compliance with preoperative laboratory testing was good, with 94.3% of nutritional assessments and 91.4% of obesity-related comorbidity assessments carried out, and in the second year following BS, this rate was 73.9%. Regarding vitamin, iron, and calcium prescriptions, the prescription rate was low. The first year was 66%, 24.9%, and 21%, and in the second year following BS, it dropped to 52.1%, 19.3%, and 11.7%, concluding that efforts should still be made to improve long-term follow-up in general and patient adherence to micronutrient supplementation in particular. This kind of quality assessment is a crucial strategy that aids in monitoring the quality of the overall assessment of these patients, and more studies in other countries, like ours, are needed [20].

In Colombia, one important factor that influences healthcare system quality and the current health system is financial distribution [108], which is regulated by two main regimens that are a right and mandatory for everyone: subsidized, made for people in vulnerable conditions, and contributory, made for people with financial capacity [109]. Additionally, persons with higher income can pay health insurance that provides access to higher-quality healthcare.

In the medical practice, inequity of these financial resources is widely seen [110], so socioeconomic status and even the type of insurance are determinant factors of the opportunity to access quality health services [110,111]. That is why although B.S. in Colombia is covered by the Mandatory Health Plan, the quality of attention will vary; for example, under some health insurance providers, the opportunity for an appointment with a general practitioner may take days, and an appointment with a specialized physician may take weeks to months. On the other hand, patients with additional health insurance can access specialized medicine in less than a week, and the medication delivery, procedure scheduling, and response times are considerably faster. All of this represents a risk factor for complications in patients with many conditions, such as post-bariatric patients [112].

## 6. Conclusions

These cases were a detailed description of the course of the neurologic, cognitive, and psychiatric symptoms of three patients with TD after bariatric surgery, highlighting the profound impact of late diagnosis and treatment. The main initial symptoms were encephalopathy and peripheral neuropathy, and the most impactful sequelae were cognitive symptoms such as executive functioning, memory, visuospatial abilities, and psychiatric manifestations, mainly depression and anxiety.

B.S. has become a highly effective intervention for the treatment of obesity, with well-known benefits after weight loss. However, it is important to understand that the pre-surgical preparation, post-surgical care, and patient education are equally as important as the surgery itself, to avoid the devastating and sometimes irreversible sequelae of micronutrient deficiency.

TD-related conditions are preventable and should be suspected and treated promptly. There is a need to raise awareness regarding the significance of this disease and its devastating consequences. The bariatric surgery team, including bariatric surgeons, postoperative care nurses, and emergency room physicians, should develop effective preventive strategies and closely monitor these patients to reduce the incidence of this disease.

Future efforts should focus on improving the knowledge of TD in the medical community, optimizing preoperative protocols, standardizing postoperative management guidelines, and enhancing healthcare provider awareness to ensure optimal outcomes for patients undergoing bariatric surgery.

## Figures and Tables

**Table 1:** Socio-demographic characteristics of the patients.

Characteristics	Patient 1	Patient 2	Patient 3
Sex	Female	Female	Female
Age	30	22	28
Profession	Hairdresser	Lawyer	None
Comorbidities	Amenorrhea	No	No
History of alcoholism	No	No	No
History of psychiatric disorders	No	No	Personality disorder
Baseline body mass index (kg/m^2^)	43	43.3	34.9

**Table 2:** Clinical findings and their progression.

Clinical Features	Initial Findings			On Discharge from the Hospital			>1 Year after Diagnosis		
	Case 1	Case 2	Case 3	Case 1	Case 2	Case 3	Case 1	Case 2	Case 3
Encephalopathy	✓	✓	✓	---	---	---	---	---	---
Anterograde amnesia	Severe	Severe	Severe	Severe	Severe	Severe	Slight	Severe	Severe
Ophthalmoplegia	✓	---	---	---	---	---	---	---	---
Nystagmus	Multidirectional	Vertical	Multidirectional	Multidirectional	Vertical	Multidirectional	Multidirectional	Vertical	Multidirectional
Weakness	UEs 3/5	UEs 5/5	UEs 5/5	UEs 3/5	UEs 5/5	UEs 5/5	UEs 5/5	UEs 5/5	MMSS 5/5 MMII 4/5 proximal, 3/5 distal
	LEs 2/5	LEs 1/5	LEs 2/5	ILE 2/5	LEs 1/5	LEs 3/5	LEs 4/5	LEs 4+/5	
Positive sensory symptoms	Allodynia	Allodynia	---	---	Allodynia	---	---	Continuous urgent pain	---
Negative sensory symptoms	---	---	Appalesthesia	---	---	Appalesthesia	---	---	---
Reduced reflexes	Areflexia	Areflexia	Areflexia	Areflexia	Areflexia	Areflexia	Areflexia	Areflexia	Areflexia
Other symptoms	---	Significant hearing loss, vertigo, and tinnitus	Tinnitus	---	Significant hearing loss, vertigo, and tinnitus	---	---	Moderate hearing loss, vertigo, and tinnitus	---

UEs: upper extremities; LEs: lower extremities.

**Table 3:** Diagnostic findings and thiamine dosage.

Clinical Information	Patient 1	Patient 2	Patient 3
Treatment	Thiamine, 500 mg diluted in 100 mL of dextrose 5% in water to be administered intravenously every 8 h over 30 min for three days, followed by 250 mg every 8 h for five days, then 100 mg orally every 8 h for 15 days, and finally continue 100 mg P.O. every 24 h for one year.	Thiamine, 500 mg diluted in 100 mL of 5% in water, to be administered intravenously every 8 h in 30 min for two weeks and continue 100 mg P.O. every day for one year.	Thiamine 500 mg diluted in 100 mL of 0.9% sodium chloride solution to be given IV every 8 h for 30 min for five days, followed by 300 mg every 3 h for two months and then indefinitely.
Neuroimaging for W.D.	Typical	Typical	Typical
Serum thiamine levels by high-performance liquid chromatography DV: 2.8–8.5	19 (12 September 2021, on day nine of thiamine treatment)	28	3.6
Nerve conduction studies	Sensory–motor polyneuropathy with myelin and axonal involvement of moderate to severe intensity in all four extremities.	Sensory–motor polyneuropathy with severe myelin and axonal involvement of the lower limbs.	Sensory–motor polyneuropathy with severe myelin and axonal involvement of the lower limbs.

**Table 4:** Neuropsychological findings exhibited during patient evaluation.

	CASE 1			CASE 2			CASE 3		
TEST	PD	PER	PT	PD	PER	PT	PD	PER	PT
HVLT-A	16	25	44–45	11	5	34–37	18	40	48–49
HVLT-B	7	55	52–55	0	0	0	0	0	0
TMT-A	34	95	61	31	95	61	60	65	48–49
TMT-B	134	60	46–47	0	0	0	140	55	45
STROOP P	107	80	59	0	0	0	102	80	59
STROOP C	77	85	60–62	60	55	52	72	80	58–59
STROOP P-C	50	90	63–67	0	0	0	36	60	52
FVS-S	19	75	57–58	22	90	63–66	17	60	53–54
FAS-F	14	80	60–63	14	80	60–63	12	70	55–56
FAS-A	11	55	51–52	16	80	60–63	6	15	40–41
FAS-S	9	45	48–49	12	70	55–56	12	70	55–56
BVRT	60	>95	65–68	60	>95	65–68	60	>95	65–68
SDMT	62	>95	69–77	58	95	68	27	50	48–49
RCFT-A	36	85	60	36	85	60	30	45	52
RCFT-B	28	90	64–67	12	40	46–47	8	20	40–42

TMT: Trail Making Test; STROOP P: Stroop test word: STROOP C: Stroop test color; STROOP P-C: Stroop test word and color; HVLT: Hopkins Verbal Learning Test; FVS: semantic verbal fluency task; FAS: phonological verbal fluency test; SDMT: digits and symbols test; Rey complex figure Test; BVRT: Boston Verbal Learning Test; PD: direct score; PER: percentile score; PT: T-score.

**Table 5:** The operational criteria for the clinical diagnosis of WE, as proposed by Caine et al. [88].

Symptom or Sign	As Evidenced by One or More of the Following
Dietary deficiencies	Undernutrition (body mass index < 2 S.D. below normal) A history of grossly impaired dietary intake An abnormal thiamine status
Oculomotor abnormalities	Ophthalmoplegia Nystagmus Gaze palsy
Cerebellar dysfunction	Unsteadiness or ataxia Abnormalities of past pointing Dysdiadokokinesia Impaired heel-to-shin test
Either an altered mental state or mild memory impairment	Disorientation in two of three fields: Confusion An abnormal digit span test Comatose Or Failure to remember two or more words in a four-item memory test; Impairment in more elaborate neuropsychological tests of memory function.

Note: When two of these four criteria apply, the clinical diagnosis of WE is made. These criteria have a lower sensitivity in the context of hepatic encephalopathy. Abbreviation: WE, Wernicke encephalopathy.

## Data Availability

All data underlying the results are available in the article; no additional source data are required.

## References

[1] Semba R.D. (2012). The discovery of the vitamins. Int. J. Vitam. Nutr. Res..

[2] Nebel J.F.K. (2018). The Discovery of Vitamins: A History.

[3] Shoemaker D.J. (2019). The Story of Vitamins: A History of the Quest to Understand and Fight Disease.

[4] Uschner W.O., Noeske J., Tanner M. (2009). Deficiency diseases from a global perspective. Bull. World Health Organ..

[5] McCormick W.J. (1974). The conquest of beriberi. Lancet.

[6] Klein R.I., Mitchell S.L. (2004). Rickets in the United States: A century of progress. Pediatrics.

[7] Jiang Z., Pu R., Li N., Chen C., Li J., Dai W., Yang G. (2023). High prevalence of vitamin D deficiency in Asia: A systematic review and meta-analysis. Crit. Rev. Food Sci. Nutr..

[8] Pérez-López F.R., Pasupuleti V., Mezones-Holguin E., Benites-Zapata V.A., Thota P., Deshpande A., Hernandez A.V. (2015). Effect of vitamin D supplementation during pregnancy on maternal and neonatal outcomes: A systematic review and meta-analysis of randomized controlled trials. Fertil. Steril..

[9] Cai H., Wang X., Li J., Wang S., Wang L. (2021). Diuretics and micronutrients: A review of the evidence. Nutrients.

[10] Gómez-Martínez L., García-Rodríguez C., García-Hernández A., Soriano-Gómez J., López-Mármol A., Rodríguez-Rodríguez J.M. (2019). Micronutrient deficiencies in patients receiving parenteral nutrition: A systematic review and meta-analysis. Nutrients.

[11] Deng Z., Zhang Z., Li J., Li S., Wang L. (2022). Micronutrient deficiencies in patients with chronic kidney disease taking diuretics: A systematic review and meta-analysis. Nutrients.

[12] Csendes, Araya J., Diaz E., de Valdes O.D., Smok G., Lopez F. (2013). Dietary and nutritional aspects of bariatric surgery: A review of the literature. Obes. Surg..

[13] Phelps S., Whitlock K.D., Johnson C.D., Kahan S., Peterson K.M., Miller K.K. (2015). The association between bariatric surgery and thiamine deficiency: A systematic review and meta-analysis. Obes. Surg..

[14] Oliveira, Ribeiro A.B., Silva A.A., Cardoso M.A., Silva A.P., Moura A.A. (2020). Prevalence of thiamine deficiency after Roux-en-Y gastric bypass: A systematic review and meta-analysis. Obes. Surg..

[15] Tang L., Alsulaim H.A., Canner J.K., Prokopowicz G.P., Steele K.E. (2018). Prevalence and predictors of postoperative thiamine deficiency after vertical sleeve gastrectomy. Surg. Obes. Relat. Dis..

[16] Behnagh K., Eghbali M., Abdolmaleki F., Abbasi M., Mottaghi A. (2024). Pre-and Post-Surgical Prevalence of Thiamine Deficiency in Patients Undergoing Bariatric Surgery: A Systematic Review and Meta-Analysis. Obes. Surg..

[17] Guillerme S., Delarue J., Thereaux J. (2023). Clinical pathways in the management of the obese: Pre- and postoperative aspects. J. Visc. Surg. (JVS).

[18] Hsu J.L., Farrell T. (2024). Updates in Bariatric Surgery. Am. J. Surg..

[19] Verras G.I., Mulita F., Parmar C., Drakos N., Bouchagier K., Kaplanis C., Skroubis G. (2023). Outcomes at 10-Year Follow-Up after Roux-en-Y Gastric Bypass, Biliopancreatic Diversion, and Sleeve Gastrectomy. J. Clin. Med..

[20] Najah H., Duffillot C., Gronnier C., Lescarret B., Saubusse E., Collet D., Gatta-Cherifi B., Montsaingeon-Henry M. (2022). Guideline compliance in bariatric surgery: A French nationwide study. Surg. Obes. Relat. Dis..

[21] Spetz K., Hult M., Olbers T., Bonn S., Svedjeholm S., Lagerros Y.T., Andersson E. (2022). A smartphone application to improve adherence to vitamin and mineral supplementation after bariatric surgery. Obesity.

[22] Nguyen N.T., Varela J.E. (2017). Bariatric surgery for obesity and metabolic disorders: State of the art. Nat. Rev. Gastroenterol. Hepatol..

[23] Vecino-Ortiz A.I., Arroyo-Ariza D. (2018). A tax on sugar sweetened beverages in Colombia: Estimating the impact on overweight and obesity prevalence across socio economic levels. Soc. Sci. Med..

[24] Jimenez-Mora M.A., Nieves-Barreto L.D., Montaño-Rodríguez A., Betancourt-Villamizar E.C., Mendivil C.O. (2020). Association of Overweight, Obesity and Abdominal Obesity with Socioeconomic Status and Educational Level in Colombia. Diabetes Metab. Syndr. Obes..

[25] Fruh S.M. (2017). Obesity: Risk factors; complications, and strategies for sustainable long-term weight management. J. Am. Assoc. Nurse Pract..

[26] Maciejewski M.L., Arterburn D., Van Scoyoc L., Smith V.A., Yancy W.S., Weidenbacher H.J., Livingston E.H., Olsen M.K. (2016). Bariatric surgery and long-term durability of weight loss. JAMA Surg..

[27] Nguyen N.T., Slone J.A., Nguyen X.M.T., Hartman J.S., Hoyt D.B. (2009). A prospective randomized trial of laparoscopic gastric bypass versus laparoscopic adjustable gastric banding for the treatment of morbid obesity: Outcomes, quality of life, and costs. Ann. Surg..

[28] Ballantyne G.H. (2003). Measuring outcomes following bariatric surgery: Weight loss parameters, improvement in co-morbid conditions, change in quality of life and patient satisfaction. Obes. Surg..

[29] Wilhelm S.M., Young J., Kale-Pradhan P.B. (2014). Effect of bariatric surgery on hypertension: A meta-analysis. Ann. Pharmacother..

[30] Kuno T., Tanimoto E., Morita S., Shimada Y.J. (2019). Effects of bariatric surgery on cardiovascular disease: A concise update of recent advances. Front. Cardiovasc. Med..

[31] Boido A., Ceriani V., Cetta F., Lombardi F., Pontiroli A.E. (2015). Bariatric surgery and prevention of cardiovascular events and mortality in morbid obesity: Mechanisms of action and choice of surgery. Nutr. Metab. Cardiovasc. Dis..

[32] Khorgami Z., Shoar S., Saber A.A., Howard C.A., Danaei G., Sclabas G.M. (2019). Outcomes of bariatric surgery versus medical management for type 2 diabetes mellitus: A meta-analysis of randomized controlled trials. Obes. Surg..

[33] Wu T., Wong S.K.H., Law B.T.T., Grieve E., Wu O., Tong D.K.H., Wong C.K.H. (2020). Five-year effectiveness of bariatric surgery on disease remission, weight loss, and changes of metabolic parameters in obese patients with type 2 diabetes: A population-based propensity score-matched cohort study. Diabetes/Metab. Res. Rev..

[34] Inabnet W.B., Winegar D.A., Sherif B., Sarr M.G. (2012). Early outcomes of bariatric surgery in patients with metabolic syndrome: An analysis of the bariatric outcomes longitudinal database. J. Am. Coll. Surg..

[35] Möller F., Hedberg J., Skogar M., Sundbom M. (2023). Long-term follow-up 15 years after duodenal switch or gastric bypass for super obesity: A randomized controlled trial. Obes. Surg..

[36] Li M., Song J.R., Zhao J., Wang C.F., Zhang C.S., Wang H.D., Dong J. (2022). The effects of bariatric surgery on cognition in patients with obesity: A systematic review and meta-analysis. Surg. Obes. Relat. Dis. Off. J. Am. Soc. Bariatr. Surg..

[37] Ota Y., Capizzano A.A., Moritani T., Naganawa S., Kurokawa R., Srinivasan A. (2020). Comprehensive review of Wernicke encephalopathy: Pathophysiology, clinical symptoms and imaging findings. Jpn. J. Radiol..

[38] Piché M.E., Tchernof A., Després J.P. (2020). Obesity phenotypes, diabetes, and cardiovascular diseases. Circ. Res..

[39] Alabduljabbar K., Bonanos E., Miras A.D., le Roux C.W. (2023). Mechanisms of Action of Bariatric Surgery on Body Weight Regulation. Gastroenterol. Clin..

[40] Lefere S., Onghena L., Vanlander A., Van Nieuwenhove Y., Devisscher L., Geerts A. (2021). Bariatric surgery and the liver—Mechanisms, benefits, and risks. Obes. Rev..

[41] Lupoli R., Lembo E., Saldalamacchia G., Avola C.K., Angrisani L., Capaldo B. (2017). Bariatric surgery and long-term nutritional issues. World J. Diabetes.

[42] Elder K.A., Wolfe B.M. (2007). Bariatric surgery: A review of procedures and outcomes. Gastroenterology.

[43] Dagan S.S., Goldenshluger A., Globus I., Schweiger C., Kessler Y., Kowen Sandbank G., Sinai T. (2017). Nutritional recommendations for adult bariatric surgery patients: Clinical practice. Adv. Nutr..

[44] Pellegrini M., Rahimi F., Boschetti S., Devecchi A., De Francesco A., Mancino M.V., Bo S. (2021). Pre-operative micronutrient deficiencies in patients with severe obesity candidates for bariatric surgery. J. Endocrinol. Investig..

[45] Patel R., Saumoy M. (2021). Treatment of Micronutrient Deficiencies Pre and Post Bariatric Surgery. Curr. Treat. Options Gastroenterol..

[46] Aasheim T., Björkman S., Søvik T.T., Engström M., Hanvold S.E., Mala T., Bøhmer T. (2009). Vitamin status after bariatric surgery: A randomized study of gastric bypass and duodenal switch. Am. J. Clin. Nutr..

[47] Zhang J., Liu Y. (2017). Theoretical study of the catalytic mechanism of glyoxylate carboligase and its mutant V51E. Theor. Chem. Acc..

[48] Moreland J.L. (1980). The role of transketolase in cellular metabolism. Am. J. Clin. Nutr..

[49] Utter M.F., Scrutton M.C. (1967). The regulation of phosphoenolpyruvate carboxylase by magnesium and other divalent metal ions. J. Biol. Chem..

[50] Chou A., Clomburg J.M., Qian S., Gonzalez R. (2019). 2-Hydroxyacyl-CoA lyase catalyzes acyloin condensation for one-carbon bioconversion. Nat. Chem. Biol..

[51] Foulon V., Antonenkov V.D., Croes K., Waelkens E., Mannaerts G.P., Van Veldhoven P.P., Casteels M. (1999). Purification, molecular cloning, and expression of 2-hydroxyphytanoyl-CoA lyase, a peroxisomal thiamine pyrophosphate-dependent enzyme that catalyzes the carbon–carbon bond cleavage during α-oxidation of 3-methyl-branched fatty acids. Proc. Natl. Acad. Sci. USA.

[52] Brosnan J.T., Brosnan M.E. (2006). Branched-chain amino acids: Metabolism and the brain. J. Nutr..

[53] Nakata K., Kashiwagi T., Kunishima N., Naitow H., Matsuura Y., Miyano H., Iwata S. (2023). Ambient temperature structure of phosphoketolase from Bifidobacterium longum determined by serial femtosecond X-ray crystallography. Acta Crystallogr. Sect. D Struct. Biol..

[54] Chipman D., Barak Z.E., Schloss J.V. (1998). Biosynthesis of 2-aceto-2-hydroxy acids: Acetolactate synthases and acetohydroxyacid synthases. Biochim. Biophys. Acta (BBA)-Protein Struct. Mol. Enzymol..

[55] Paulikat M., Wechsler C., Tittmann K., Mata R.A. (2017). Theoretical studies of the electronic absorption spectra of thiamin diphosphate in pyruvate decarboxylase. Biochemistry.

[56] Mkrtchyan G., Graf A., Bettendorff L., Bunik V. (2016). Cellular thiamine status is coupled to function of mitochondrial 2-oxoglutarate dehydrogenase. Neurochem. Int..

[57] Graupner M., Xu H., White R. (2000). Identification of the gene encoding sulfopyruvate decarboxylase, an enzyme involved in biosynthesis of coenzyme M. J. Bacteriol..

[58] Chen P.Y.T., Aman H., Can M., Ragsdale S.W., Drennan C.L. (2018). Binding site for coenzyme A revealed in the structure of pyruvate: Ferredoxin oxidoreductase from Moorella thermoacetica. Proc. Natl. Acad. Sci. USA.

[59] Asakawa T., Wada H., Yamano T. (1968). Enzymatic conversion of phenylpyruvate to phenylacetate. Biochim. Biophys. Acta (BBA)-Gen. Subj..

[60] Begley T.P., Downs D.M., Ealick S.E., McLafferty F.W., Van Loon A.P., Taylor S., Xi J. (1999). Thiamin biosynthesis in prokaryotes. Arch. Microbiol..

[61] Kritikos G., Parr J.M., Verbrugghe A. (2017). The role of thiamine and effects of deficiency in dogs and cats. Vet. Sci..

[62] Tylicki A., Łotowski Z., Siemieniuk M., Ratkiewicz A. (2018). Thiamine and selected thiamine antivitamins—Biological activity and methods of synthesis. Biosci. Rep..

[63] Gangolf M., Czerniecki J., Radermecker M., Detry O., Nisolle M., Jouan C., Bettendorff L. (2010). Thiamine status in humans and content of phosphorylated thiamine derivatives in biopsies and cultured cells. PLoS ONE.

[64] Bijarnia-Mahay S., Saini A.G., Mahay H.S. (2023). Thiamine, transporters, and epilepsy. Vitamins and Minerals in Neurological Disorders.

[65] Said M., Balamurugan K., Subramanian V.S., Marchant J.S. (2004). Expression and functional contribution of hTHTR-2 in thiamin absorption in human intestine. Am. J. Physiol.-Gastrointest. Liver Physiol..

[66] Mrowicka M., Mrowicki J., Dragan G., Majsterek I. (2023). The importance of thiamine (vitamin B1) in humans. Biosci. Rep..

[67] Tallaksen M.E., Sande A., Bøhmer T., Bell H., Karlsen J. (1993). Kinetics of thiamin and thiamin phosphate esters in human blood, plasma and urine after 50 mg intravenously or orally. Eur. J. Clin. Pharmacol..

[68] Bunik V.I., Tylicki A., Lukashev N.V. (2013). Thiamin diphosphate-dependent enzymes: From enzymology to metabolic regulation, drug design and disease models. FEBS J..

[69] Strumilo S. (2005). Often ignored facts about the control of the 2-oxoglutarate dehydrogenase complex. BAMBED.

[70] Zhao J., Zhong C.J. (2009). A reviev on research progress of transketolase. Neurosci. Bull..

[71] Bunik V.I., Strumilo S. (2009). Regulation of catalysis within cellular network: Metabolic and signaling implications of the 2-oxoglutarate oxidative decarboxylation. Curr. Chem. Biol..

[72] Bettendorff L., Wins P. (2009). Thiamin diphosphate in biological chemistry: New aspects of thiamin metabolism, especially triphosphate derivatives acting other than as cofactors. FEBS J..

[73] Lukienko P.I., Mel N.G., Zverinskii I.V., Zabrodskaya S.V. (2000). Antioxidant properties of thiamine. Bull. Exp. Biol. Med..

[74] Wang C., Liang J., Zhang C., Bi Y., Shi X., Shi Q. (2007). Effect of ascorbic acid and thiamine supplementation at different concentrations on lead toxicity in liver. Ann. Occup. Hyg..

[75] Whitfield C., Bourassa M.W., Adamolekun B., Bergeron G., Bettendorff L., Brown K.H., Combs G.F. (2018). Thiamine deficiency disorders: Diagnosis; prevalence, and a roadmap for global control programs. Ann. N. Y. Acad. Sci..

[76] Dhir S., Tarasenko M., Napoli E., Giulivi C. (2019). Neurological; psychiatric, and biochemical aspects of thiamine deficiency in children and adults. Front. Psychiatry.

[77] Sechi G., Serra A. (2007). Wernicke’s encephalopathy: New clinical settings and recent advances in diagnosis and management. Lancet Neurol..

[78] Kröll D., Laimer M., Borbély Y.M., Laederach K., Candinas D., Nett P.C. (2016). Wernicke encephalopathy: A future problem even after sleeve gastrectomy? A systematic literature review. Obes. Surg..

[79] Gomes F., Bergeron G., Bourassa M.W., Fischer P.R. (2021). Thiamine deficiency unrelated to alcohol consumption in high-income countries: A literature review. Ann. N. Y. Acad. Sci..

[80] Sinha S., Kataria A., Kolla B.P., Thusius N., Loukianova L.L. (2019). Wernicke encephalopathy—Clinical pearls. Mayo Clin. Proc..

[81] Williams R.D., Mason H.L., Wilder R.M., Smith B.F. (1940). Observations on induced thiamine (vitamin B1) deficiency in man. Arch. Intern. Med..

[82] Prakash S. (2018). Gastrointestinal beriberi: A forme fruste of Wernicke’s encephalopathy?. Case Rep..

[83] Kohnke S., Meek C.L. (2021). Don’t seek, don’t find: The diagnostic challenge of Wernicke’s encephalopathy. Ann. Clin. Biochem..

[84] Habas E., Farfar K., Errayes N., Rayani A., Elzouki A.N. (2023). Wernicke Encephalopathy: An Updated Narrative Review. Saudi J. Med. Med. Sci..

[85] Chandrakumar A., Bhardwaj A., Jong G.W.T. (2019). Review of thiamine deficiency disorders: Wernicke encephalopathy and Korsakoff psychosis. J. Basic Clin. Physiol. Pharmacol..

[86] Marrs C., Lonsdale D. (2021). Hiding in plain sight: Modern thiamine deficiency. Cells.

[87] Harper G., Giles M., Finlay-Jones R. (1986). Clinical signs in the Wernicke-Korsakoff complex: A retrospective analysis of 131 cases diagnosed at necropsy. J. Neurol. Neurosurg. Psychiatry.

[88] Caine D., Halliday G.M., Kril J.J., Harper C. (1997). Operational criteria for the classification of chronic alcoholics: Identification of Wernicke’s encephalopathy. J. Neurol. Neurosurg. Psychiatry.

[89] Arts N.J., Walvoort S.J., Kessels R.P. (2017). Korsakoff’s syndrome: A critical review. Neuropsychiatr. Dis. Treat..

[90] Sharp S., Wilson M.P., Nordstrom K. (2016). Psychiatric Emergencies for Clinicians: Emergency Department Management of Wernicke-Korsakoff Syndrome. J. Emerg. Med..

[91] Lewis A., de Jersey S., Hopkins G., Hickman I., Osland E. (2018). Does bariatric surgery cause vitamin A, B1, C or E deficiency? A systematic review. Obes. Surg..

[92] Roust R., DiBaise J.K. (2017). Nutrient deficiencies prior to bariatric surgery. Curr. Opin. Clin. Nutr. Metab. Care.

[93] Kerns J.C., Arundel C., Chawla L.S. (2015). Thiamin deficiency in people with obesity. Adv. Nutr..

[94] Edwards A., Tu-Maung N., Cheng K., Wang B., Baeumner A.J., Kraft C.E. (2017). Thiamine assays—Advances, challenges, and caveats. ChemistryOpen.

[95] Jung Y.C., Chanraud S., Sullivan E.V. (2012). Neuroimaging of Wernicke’s encephalopathy and Korsakoff’s syndrome. Neuropsychol. Rev..

[96] Wijnia J.W. (2022). A Clinician’s View of Wernicke-Korsakoff Syndrome. J. Clin. Med..

[97] Johnson J.M., Fox V. (2018). Beyond thiamine: Treatment for cognitive impairment in Korsakoff’s syndrome. Psychosomatics.

[98] Nishimoto A., Usery J., Winton J.C., Twilla J. (2017). High-dose parenteral thiamine in treatment of Wernicke’s encephalopathy: Case series and review of the literature. In Vivo.

[99] Ciobârcă M., Cătoi A.F., Copăescu C., Miere D., Crişan G. (2022). Nutritional status prior to bariatric surgery for severe obesity: A review. Med. Pharm. Rep..

[100] Lorenzo D., Antoniou S.A., Batterham R.L., Busetto L., Godoroja D., Iossa A., Carrano F.M., Agresta F., Alarçon I., Azran C. (2020). Clinical practice guidelines of the European Association for Endoscopic Surgery (EAES) on bariatric surgery: Update 2020 endorsed by IFSO-EC, EASO and ESPCOP. Surg. Endosc..

[101] Mechanick J.I., Apovian C., Brethauer S., Garvey W.T., Joffe A.M., Kim J., Kushner R.F., Lindquist R., Pessah-Pollack R., Seger J. (2019). Clinical practice guidelines for the perioperative nutrition, metabolic, and nonsurgical support of patients undergoing bariatric procedures—2019 update: Cosponsored by american association of clinical endocrinologists. Endocr. Pract..

[102] Van Wissen J., Bakker N., Doodeman H.J., Jansma E.P., Bonjer H.J., Houdijk A.P. (2016). Preoperative Methods to Reduce Liver Volume in Bariatric Surgery: A Systematic Review. Obes. Surg..

[103] Gómez L.E.L., Báez G.A.S., Ramirez Leal M.P., Domínguez-Alvarado G.A. (2023). Bariatric and metabolic surgery—In the health benefits plan in Colombia. Obes. Surg..

[104] Oudman E., Wijnia J.W., van Dam M., Biter L.U., Postma A. (2018). Preventing Wernicke encephalopathy after bariatric surgery. Obes. Surg..

[105] Bettini S., Belligoli A., Fabris R., Busetto L. (2020). Diet approach before and after bariatric surgery. Rev. Endocr. Metab. Disord..

[106] Martínez-Ortega J., Olveira G., Pereira-Cunill J.L., Arraiza-Irigoyen C., García-Almeida J.M., Rocamora J.A.I., Molina-Puerta M.J., Soria J.B.M., Rabat-Restrepo J.M., Rebollo-Pérez M.I. (2020). Recommendations Based on Evidence by the Andalusian Group for Nutrition Reflection and Investigation (GARIN) for the Pre- and Postoperative Management of Patients Undergoing Obesity Surgery. Nutrients.

[107] O’Kane M., Parretti H.M., Pinkney J., Welbourn R., Hughes C.A., Mok J., Walker N., Thomas D., Devin J., Coulman K.D. (2020). British Obesity and Metabolic Surgery Society Guidelines on perioperative and postoperative biochemical monitoring and micronutrient replacement for patients undergoing bariatric surgery-2020 update. Obes. Rev. Off. J. Int. Assoc. Study Obes..

[108] Armada F., Muntaner C., Navarro V. (2001). Health and social security reforms in Latin America: The convergence of the World Health Organization, the World Bank, and Transnacional Corporations. Int. J. Health Serv..

[109] Londoño J.L., Nieto L.E. (2001). Factores socio económicos y aseguramiento en salud en el área urbana de Colombia. Rev. Fac. Nac. Salud Pública.

[110] World Health Organization (2010). A Conceptual Framework for Action on the Social Determinants of Health.

[111] Lillie-Blanton M., Hoffman C. (2005). The role of health insurance coverage in reducing racial/ethnic disparities in health care. Health Aff..

[112] Houghton N., Bascolo E., Del Riego A. (2020). Socioeconomic inequalities in access barriers to seeking health services in four Latin American countries. Rev. Panam. Salud Publica.

